# Distribution of DNA replication proteins in Drosophila cells

**DOI:** 10.1186/1471-2121-8-42

**Published:** 2007-10-15

**Authors:** Hariharan P Easwaran, Heinrich Leonhardt, M Cristina Cardoso

**Affiliations:** 1 Max Delbrück Center for Molecular Medicine, 13125 Berlin, Germany; 2 Ludwig Maximilians University Munich, Department of Biology, 82152 Planegg-Martinsried, Germany; 3 Current address : Cancer Research Building, 1650 Orleans Street, Baltimore, MD 21231-1000, USA

## Abstract

**Background:**

DNA replication in higher eukaryotic cells is organized in discrete subnuclear sites called replication foci (RF). During the S phase, most replication proteins assemble at the RF by interacting with PCNA via a PCNA binding domain (PBD). This has been shown to occur for many mammalian replication proteins, but it is not known whether this mechanism is conserved in evolution.

**Results:**

Fluorescent fusions of mammalian replication proteins, Dnmt1, HsDNA Lig I and HsPCNA were analyzed for their ability to target to RF in Drosophila cells. Except for HsPCNA, none of the other proteins and their deletions showed any accumulation at RF in Drosophila cells. We hypothesized that in Drosophila cells there might be some other peptide sequence responsible for targeting proteins to RF. To test this, we identified the DmDNA Lig I and compared the protein sequence with HsDNA Lig I. The two orthologs shared the PBD suggesting a functionally conserved role for this domain in the Drosophila counterpart. A series of deletions of DmDNA Lig I were analyzed for their ability to accumulate at RF in Drosophila and mammalian cells. Surprisingly, no accumulation at RF was observed in Drosophila cells, while in mammalian cells DmDNA Lig I accumulated at RF via its PBD. Further, GFP fusions with the PBD domains from Dnmt1, HsDNA Lig I and DmDNA Lig I, were able to target to RF only in mammalian cells but not in Drosophila cells.

**Conclusion:**

We show that S phase in Drosophila cells is characterized by formation of RF marked by PCNA like in mammalian cells. However, other than PCNA none of the replication proteins and their deletions tested here showed accumulation at RF in Drosophila cells while the same proteins and deletions are capable of accumulating at RF in mammalian cells. We hypothesize that unlike mammalian cells, in Drosophila cells, replication proteins do not form long-lasting interactions with the replication machinery, and rather perform their functions via very transient interactions at the RF.

## Background

In higher eucaryotes DNA replication progresses in a defined spatio-temporal pattern. Specific segments of the DNA/chromatin replicate as discrete regions, which when visualized by light microscopy, are called replication foci (RF). It is suggested that each replication focus is constituted of a subchromosomal domain of adjacent replicons from the same chromosomal region that replicate together in time and space and their associated protein machines (replisomes). These subchromosomal domains are maintained as single units through multiple rounds of cell division, and they occupy stable positions in the nucleus [1-4]. The existence of various patterns of RF has been demonstrated in living mammalian cells by labeling the RF with fluorescent proteins fused to core replication factors, like DNA Ligase I or PCNA [5-8]. Such studies have shown that replication proteins continuously assemble at many subchromosomal domains to form RF, and continuously disassemble from sites that have completed replication (reviewed in [9]. This cycle of assembly and disassembly progresses throughout S phase giving rise to the distinct patterns of RF.

For an understanding of the process of DNA replication as it occurs in vivo, it is essential to define the mechanisms by which various proteins assemble at the RF. The first protein identified at these foci during S phase was PCNA [10,11]. PCNA forms a homotrimeric ring around the DNA and functions as a processivity factor for the replicative DNA polymerases [12,13]. Many other proteins have since been shown to be accumulated at these sites, including the maintenance DNA methyltransferase (Dnmt1) [14], DNA polymerase alpha [15], DNA Ligase I [16,5], RPA70 and cell cycle regulators cyclin A and cdk2 [17], and DNA repair factors like uracil-DNA-glycosylase [18]. Such replication factors undergo dynamic re-distribution in the nucleus during S phase. In the G1 phase, most replication proteins are homogeneously distributed in the nucleoplasm. Upon entry into S phase, replication proteins form punctate patterns that co-localize with sites of active DNA synthesis. This accumulation at RF is mediated by specific peptide sequences in the replication proteins called replication foci targeting sequence (RFTS). A known mechanism of recruitment of replication factors to RF is through the interaction with PCNA, via a short amino acid motif termed PCNA binding domain (PBD). This has been observed to be a recurrent mechanism utilized by many replication factors. Over the years many proteins involved in DNA metabolism and cell cycle regulation have been shown to interact with PCNA via the canonical PBD found in Dnmt1 [19,7] and DNA Ligase I [20]. The PBD is conserved in homologous proteins from archaebacteria, yeast, worms, flies, amphibians and mammals [21]. Such conservation across different classes of organisms suggests that the recruitment of proteins to RF mediated by PBD-PCNA interaction is a mechanism conserved throughout evolution. Indeed, many features of DNA replication are conserved in higher eucaryotes. Firstly, general features of the replication process itself, like bi-directional replication fork movement, continuous leading and discontinuous lagging strand synthesis, requirement of RNA primers to start DNA synthesis are all conserved [22]. Secondly, the proteins involved in controlling DNA replication and catalyzing the process of DNA replication are conserved in diverse eucaryotes [23]. Thirdly, organization of replication into RF that follow a spatio-temporal pattern is a conserved feature in diverse eucaryotes that have been analyzed [24,25]. Other than the conservation of the PBD in homologous proteins, however, there is no experimental evidence whether the mechanisms by which replication factors accumulate at RF are conserved in evolution. We have, therefore, analyzed the ability of mammalian replication proteins to accumulate at the RF in Drosophila cells and vice versa.

## Results

### PCNA is highly conserved in S. cerevisiae, D. melanogaster and mammals

**Figure 1:**
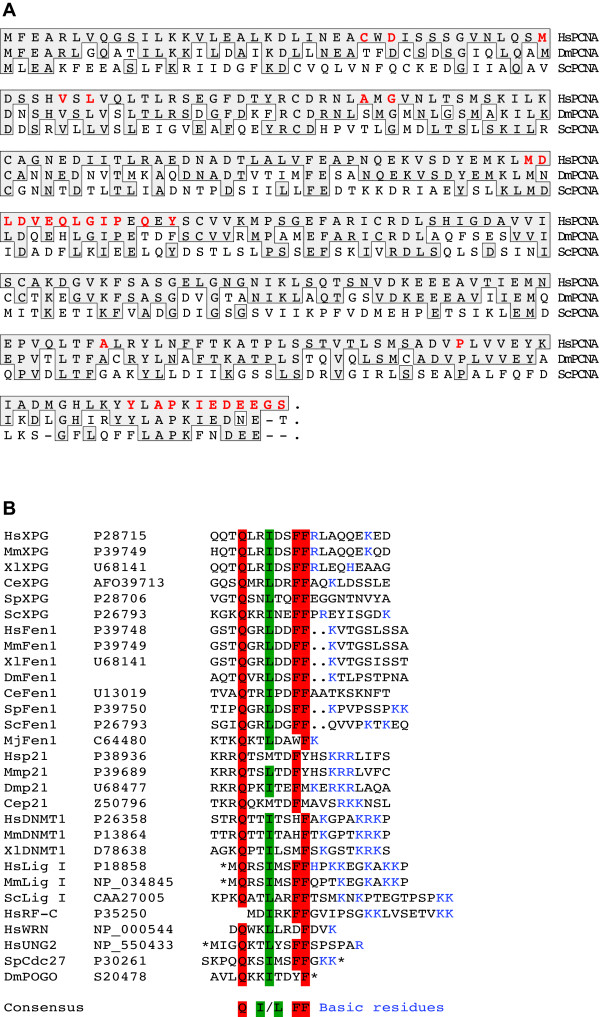
**Drosophila and human PCNA are highly similar**. (A)Protein sequence alignment of HsPCNA (human), DmPCNA (Drosophila) and ScPCNA (budding yeast). Residues identical to HsPCNA are boxed. Regions in HsPCNA that interact with the PBD of p21 [26] are in red. (B) A conserved PBD in proteins involved in DNA metabolism. Alignment of the PBD from various homologous proteins from different organisms. Accession numbers are indicated in the second column where available. Asterisk denotes the beginning or end of the protein. Identical residues are highlighted in red and conserved substitution in green. (Hs: Homo sapiens; Mm: Mus musculus; Xl: Xenopus laevis; Dm: Drosophila melanogaster; Ce: Caenorhabditis elegans; Sp: Schizosaccharomyces pombe; Sc: Saccharomyces cerevisiae; Mj: Methanococcus jannaschii).

The mechanistic basis for the association of replication factors with RF has been proposed to be mediated via interaction of PBD with PCNA. To understand whether the PBD-mediated association of proteins with RF is conserved in evolution, we first analyzed the conservation of PCNA protein sequence from divergent eucaryotes, especially the regions shown to interact with the PBD of p21_Cip1/Waf1 _[26]. The alignment in Figure 1A shows that PCNA is conserved in organisms as divergent as yeast, flies and mammals. HsPCNA is about 35% identical to ScPCNA and 70% identical to DmPCNA. The residues of PCNA that interact with the PBD of p21_Cip1/Waf1 _are more than 75% identical. Also the PBD is conserved in homologous proteins involved in DNA metabolism from archaebacteria, yeast, worms, flies, amphibians and mammals (Figure 1B) (see also [21]) suggesting that the association of proteins to RF mediated by PCNA is a mechanism conserved through evolution. We put this hypothesis to test by analyzing whether the PBD containing mammalian replication proteins can accumulate at RF in Drosophila cells.

### Human PCNA and Drosophila PCNA can accumulate at RF across the two organisms

Before testing whether replication proteins could accumulate at the RF across species and whether PCNA binding would be required, we had to first establish whether the PCNA in the two systems were interchangeable in that it could load onto RF in the heterologous system. To this end, we analyzed the association of human PCNA (HsPCNA) with RF in Drosophila cells (S2 cells) and Drosophila PCNA (DmPCNA) in mouse cells (C2C12 cells). As shown in Figure 1 both have 70% overall amino acid identity.

**Figure 2:**
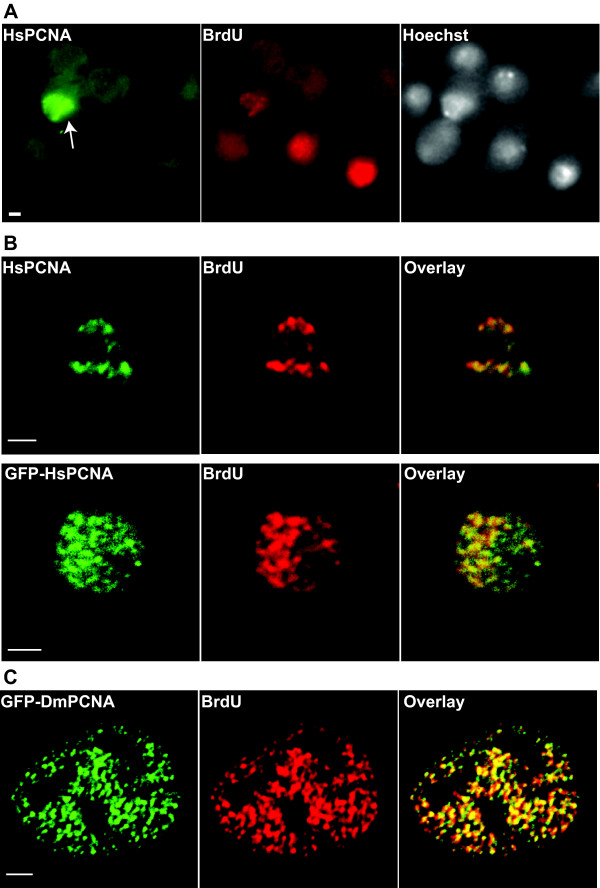
**Mammalian and Drosophila PCNA accumulate at RF interchangeably between these divergent organisms**. Drosophila S2 and mammalian C2C12 cells were transfected, pulse labeled with BrdU to label sites of active DNA replication, and fixed with formaldehyde followed by immunostaining. (A) S2 cells expressing HsPCNA and coimmunostained with anti-PCNA (FL-261; Santa Cruz) and anti-BrdU antibodies. Anti-PCNA antibody (FL-261) specifically stains transfected cell (arrow) expressing HsPCNA. (B) S2 cells expressing HsPCNA (top) or GFP-HsPCNA (bottom) and immunostained with anti-BrdU. HsPCNA was detected as in (A). Overlay shows colocalization of HsPCNA and GFP-HsPCNA with BrdU foci. (C) C2C12 cells transfected with plasmid encoding GFP-DmPCNA and immunostained with anti-BrdU. Scale bar = 2 μm.

Expression plasmids encoding HsPCNA, GFP-HsPCNA and GFP-DmPCNA were constructed and introduced into cells by calcium phosphate transfection. Subnuclear localization of HsPCNA in transfected S2 cells was accomplished by indirect immunostaining with anti-PCNA antibody (FL261, Santa Cruz) that reacted specifically with HsPCNA (Figure 2A). Transfected cells in S phase showed punctate BrdU staining representing RF to which HsPCNA colocalized (Figure 2B). Also GFP-HsPCNA is targeted to RF in S2 cells (Figure 2B). Similarly, mammalian C2C12 cells expressing GFP-DmPCNA showed complete colocalization of GFP-DmPCNA with RF (Figure 2C). Since the available antibody against DmPCNA cross reacts with mouse PCNA, localization of untagged DmPCNA in mouse cells was not possible. Owing to the high sequence similarity of HsPCNA with DmPCNA (Figure 1A) and the conserved structure of PCNA from divergent eucaryotes [27], efficient association of HsPCNA with RF in S2 cells indicates that HsPCNA must be replacing endogenous PCNA in S2 cells. Thus, these results show that the basic replication machinery in the two organisms is conserved and core replication factors are interchangeable.

It appears from the images that the size of RF in Drosophila are larger than in mammalian cells. However, measurement of the size of RF in mammalian cells showed that the average size of early/mid-S phase RF is 0.52 μm (std. deviation = 0.11) while in Drosophila the average size is 0.37 μm (std. deviation = 0.06). The contrary appears to be the case in the images because Drosophila nuclei are small and the images are presented at a higher digital zoom as can be seen from the different scale bars for mammalian and Drosophila nuclei. Further, compared to mammalian cells, there appears to be lower number of RF in Drosophila cells. This is probably due to the smaller size of the Drosophila genome (about 1/22 times the mammalian genome).

### Subnuclear localization of PCNA interacting proteins during S phase in Drosophila cells

**Figure 3:**
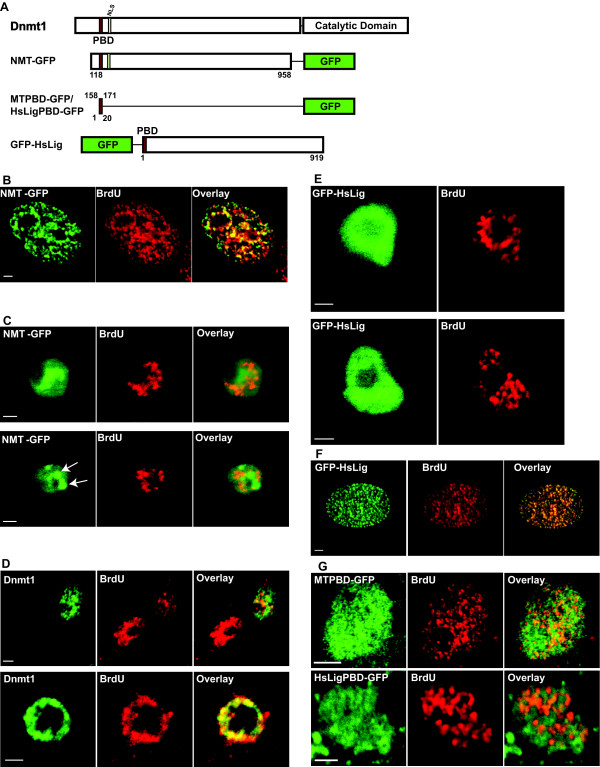
**Subnuclear localization of PCNA-interacting proteins during S phase in Drosophila cells**. (A) Plasmid constructs used to evaluate the association of mammalian replication proteins with RF in S2 cells. Mammalian cells expressing: NMT-GFP (B), GFP-HsLig (F). Drosophila cells expressing: NMT-GFP (C), Dnmt1 (D), GFP-HsLig (E), MTPBD-GFP (G-top panel) and HsLigPBD-GFP (G-bottom panel). RF were detected by BrdU incorporation and immunostaining with anti-BrdU. In (C) arrows in bottom panel show aggregation of NMT-GFP in large structures that are excluded from BrdU incorporation sites. In (D) top panel represents pattern observed in 80–90% of cells and bottom panel represents pattern observed in 10% of cells. Overlays show merge of green and red images. Scale bar = 2 μm.

In order to determine whether the mechanisms mediating recruitment of proteins to RF in different organisms are conserved, we evaluated the ability of mammalian Dnmt1 to accumulate at RF in Drosophila S2 cells. S2 cells were transfected with plasmids encoding chimeric fusions of the N-terminal domain of Dnmt1 with the GFP epitope (NMT-GFP) (summarized in Figure 3A). NMT-GFP accumulated at RF in mouse C2C12 cells (Figure 3B). NMT-GFP has a PBD and therefore it is predicted that this fusion protein should target to RF in Drosophila S2 cells. Surprisingly, NMT-GFP did not colocalize with RF in S2 cells (Figure 3C). Rather, in S2 cells, NMT-GFP seemed to be diffused throughout the nucleus as well as aggregating in some regions to form large structures. To test whether this is an artifact of the fusion protein in S2 cells, S2 cells were transfected with a plasmid encoding full length untagged Dnmt1(s) and the association of Dnmt1(s) with RF was analyzed. Like NMT-GFP, Dnmt1(s) did not accumulate at RF (Figure 3D; top panel) in the majority of cells, though in about 10% cells in S phase Dnmt1(s) seemed to partially colocalize with RF (Figure 3D; bottom panel), or with a subset of Drosophila chromatin. The latter did not correspond to the chromocenter (data not shown). The extent of colocalization of Dnmt1 and BrdU was analyzed quantitatively by calculating the Pearson's correlation coefficient (PC) and Mander's coefficient. The former gives an estimate of intensity-based correlation of the green (Dnmt1) and red (BrdU) signals. The PC value ranges from -1 (no correlation) to +1 (complete correlation) with values in between indicating different degrees of partial correlation. The average PC value (N = 10) for Dnmt1 and BrdU is 0.66 (std. deviation = 0.08) indicating that there is a partial colocalization. The Mander's coefficient measures the fraction of green (Dnmt1) signal overlapping with red (BrdU) signal and vice versa. On an average 23% (std. deviation = 9%) of Dnmt1 signal showed overlap with BrdU while 47% (std. deviation = 13%) of BrdU signal showed overlap with the Dnmt1. This indicates that there is a higher proportion of Dnmt1 that is present in regions devoid of BrdU and there is a partial colocalization of Dnmt1 and BrdU. However, a closer look at the images shows that Dnmt1 tends to accumulate in regions that are devoid of BrdU and partial colocalization with BrdU occurs in regions where the Dnmt1 signal is lower than the average.

**Figure 4:**
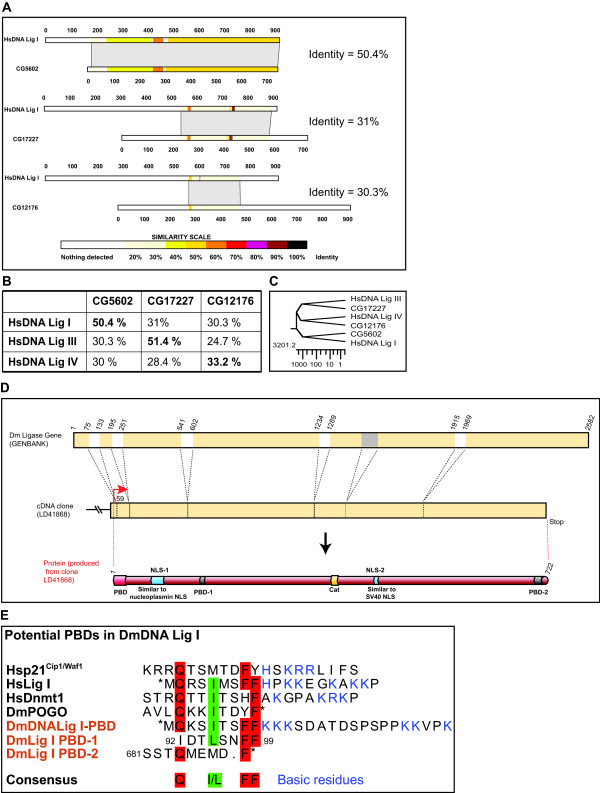
**Identification of the putative DNA Ligase I in Drosophila and its potential PBD**. BLAST search of the D. melanogaster genome and EST database with DNA Ligase I (from different species) identified three similar sequences CG5602, CG17227 and CG12176. (A) Each of the putative DNA ligase protein sequences identified in Drosophila was aligned pairwise with HsDNA Lig I. The percentage identity is indicated at the right. (B) Table showing percentage identity obtained from pairwise alignment of the three putative DNA ligase homologues in Drosophila with each of HsDNA Ligase I, III and IV. (C) Phylogenetic tree from the comparison of HsDNA ligases and the Drosophila homologues created in Lasergene program. Scale represents amino acid substitution). (D) Structure of DmDNA Lig I gene and predicted protein. The gene structure is illustrated at the top; numbers indicate nucleotide positions; white boxes represent introns. A region in the 5th exon (marked gray) was found to be absent in the cDNA clone. This region was not detected as an intron using algorithms to detect introns. In the middle the LD41868 cDNA clone obtained from ResGen. Translational start is shown by arrow and the protein produced from this cDNA clone is illustrated at the bottom. (E) PBD-like sequences identified in DmDNA Lig I protein. Asterisk indicates the beginning or end of the protein sequence. Other features as in Figure 1B.

It is surprising that a PBD-containing protein that efficiently accumulates at RF in mammalian cells does not behave similarly in Drosophila cells even though their basic replication machinery is similar. Reduced efficiency in the targeting of Dnmt1to RF in Drosophila cells could be an artifact of a protein that is foreign to Drosophila as this organism lacks a Dnmt1homologue. So we tested the targeting of human DNA Ligase I (HsDNA Lig I) to RF in S2 cells. DNA Ligase I is a core replication enzyme involved in ligating Okazaki fragments during synthesis of the lagging strand. DNA Ligase I is conserved in evolutionarily distant organisms and the Drosophila DNA Ligase I (DmDNA Lig I) homologue is 50% identical to HsDNA Lig I (Figure 4). A GFP fusion of HsDNA Lig I was expressed in S2 cells and its subnuclear localization was analyzed. GFP-HsLig showed a diffused nuclear distribution in S2 cells in all interphase stages without any discernible colocalization with RF in S phase (Figure 3E). In contrast, as shown previously [5], GFP-HsLig was efficiently targeted to RF in mouse cells (Figure 3F). Strikingly, the inability of GFP-HsLig to accumulate at RF in any of the S2 cells in S phase suggested that the PBD is unable to function as a replication foci targeting sequence (RFTS) in Drosophila. To further test this, the PBD from Dnmt1 (termed MTPBD) and HsDNA Lig I (termed HsLigPBD) were individually fused to GFP and expressed in Drosophila cells. Both MTPBD-GFP and HsLigPBD-GFP were diffusely distributed in the nucleus and did not show any discernible localization or exclusion from the RF (Figure 3G). The same fusions though were clearly targeted to RF in mammalian cells ([7] and data not shown). Thus, even though the 10 aa PBD plus the 10 aa region rich in basic residues are enough for targeting to RF as observed in mouse cells, it is unable to do so in Drosophila cells. Alternatively, in Drosophila, the mechanism behind recruitment of proteins to RF may not be dependent on the PBD, but rather some other unique protein motif might function as an RFTS.

### Search for an RFTS in Drosophila

To determine whether another protein sequence functions as an RFTS in Drosophila, we sought to clone a replication protein from Drosophila and identify its RFTS. Since DNA Ligase I is well conserved from yeast to humans, we searched the Drosophila genome database and the EST database (BDGP) [28] for putative DNA Ligase I homologues with DNA Ligase I sequence from different organisms (mouse, human, yeast) as query. Three putative homologues were identified in the EST database, viz. LD41868. AT15112, and LD06019 with corresponding predicted proteins CG5602, CG17227 and CG12176 respectively (Figure 4A). Comparison of the protein sequences of these potential homologues with HsDNA Lig I showed that the putative DNA ligase I in Drosophila is CG5602 (henceforth called DmDNA Lig I; Figure 4A–C). The EST clone (LD41868) coding for CG5602 was obtained and the sequence was verified. An 81 bp segment in the coding region of the predicted gene was found to be absent in the cDNA clone (Figure 4D). The DmDNA Lig I protein sequence was analyzed in order to identify sequences similar to the PBD. This revealed the presence of a PBD sequence (designated as DmDNA Lig I-PBD) that had all the features of HsDNA Lig I-PBD required for accumulation at replication foci, viz. the 10 amino acid region interacting with PCNA immediately followed by the 10 amino acid sequence rich in basic residues (Figure 4E). Additionally, two sequences with weak similarity to the conserved PBD were identified and called as PBD-1 and -2 (Figure 4E).

**Figure 5:**
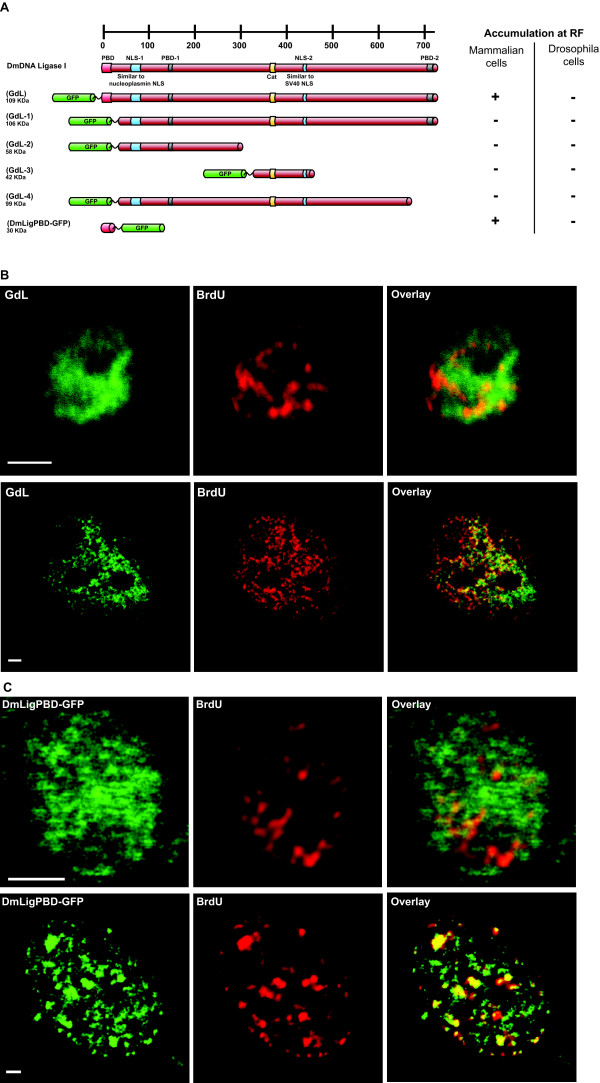
**Drosophila DNA Ligase I is targeted to RF in mouse but not in Drosophila cells**. (A) Organization of domains in DmDNA Lig I is shown at the top and below the various deletions of DmDNA Lig I fused to GFP. The predicted molecular weight of the fusion proteins is indicated on the left. The targeting to RF of each fusion in mammalian and Drosophila cells is summarized on the right. (B and C) Subcellular localization of the DmDNA Lig I (GdL, B) and DmLigPBD-GFP (C) with respect to BrdU labeled RF in S2 cells (upper panel) and C2C12 cells (bottom panel). Scale Bar = 2 μm.

To determine whether DmDNA Lig I employs some other protein sequence as an RFTS instead of the conserved PBD, a series of deletion mutants of DmDNA Lig I fused to GFP were constructed (Figure 5A) and their subnuclear localization with respect to RF was analyzed in both mouse and Drosophila cells. Immunoblotting analysis showed that proteins of the correct size were expressed from these plasmids (data not shown). The full length protein fused to GFP (GdL) did not show accumulation at RF in Drosophila S2 cells (Figure 5B), whereas it colocalized with RF in mammalian C2C12 cells (Figure 5B). None of the deletions, GdL-1, -2, -3 and -4, showed colocalization with RF in both Drosophila and mammalian cells (Figure 5A and data not shown). Thus, neither the full length DmDNA Lig I nor any of the deletions fused to GFP accumulated at RF in Drosophila cells. The fact that only GdL, but none of GdL-1 to 4 associates with RF in mouse cells indicates that this association is mediated by the DmDNA Lig I-PBD. Thus, the ability of the minimal DmDNA Lig I-PBD to accumulate at RF in Drosophila and mouse cells was analyzed. As observed with HsLigPBD-GFP and MTPBD-GFP (Figure 3G), DmLigPBD-GFP colocalized with RF in mammalian C2C12 cells but not in Drosophila S2 cells (Figure 5C). Taken together, these results show that: (i) DmDNA Lig I associates with RF in mouse cells via the DmDNA Lig I-PBD; (ii) interestingly, DmDNA Lig I does not accumulate at RF in Drosophila cells.

In summary, except for PCNA none of the other replication proteins accumulate at RF in Drosophila cells. Most notably, DmDNA Lig I does not accumulate at RF in Drosophila cells while it does so in mouse cells. This probably indicates a fundamental difference in the kinetics of association of proteins with RF in Drosophila cells and mammalian cells.

## Discussion

The observation that immunostaining for PCNA and BrdU incorporation sites gives a punctate pattern indicates that in Drosophila cells, like mammalian cells, DNA replication is organized in discrete structures or RF (Figure 2). This is consistent with the previous observation that replication in Drosophila Kc cells occurs at subnuclear foci [25]. The polymerase clamp PCNA is highly conserved between human and Drosophila (Figure 1) and HsPCNA and DmPCNA can efficiently accumulate at RF across the two organisms (Figure 2). This indicates that PCNA, a conserved central core at the replication fork, is structurally and functionally exchangeable at the RF between mammals and Drosophila. In a recent report, Kisielewska et al. showed that GFP-PCNA can be used as an S-phase marker in early stage embryo during syncytial nuclear divisions [29]. However, this study revealed that there is no punctate accumulation of GFP-HsPCNA during these early embryonic nuclear divisions. Our own unpublished results on live transgenic Drosophila embryos expressing GFP-HsPCNA corroborate these results. Inability of GFP-HsPCNA to form RF in these early cell division cycles is best explained by the short S phases resulting from firing of closely spaced origins unlike that in adult cells.

It has been proposed based on homology search data that proteins with PBD accumulate at RF in S phase cells [21]. However, in Drosophila cells PCNA interacting proteins (Dnmt1 and DNA Ligase I) did not show any accumulation at RF (Figure 3). Due to the general inability of PBD-GFP fusions from different origins (Dnmt1, HsDNA Ligase I, DmDNA Ligase I) to accumulate at RF in Drosophila cells, we argued that in Drosophila another domain might function as an RFTS. However, deletion analysis of DmDNA Ligase I fused to GFP showed that neither the full length DmDNA Ligase I nor any of the deletions accumulated at RF in Drosophila cells (Figure 5). However, DmDNA Ligase I accumulated at RF in mammalian cells via its PBD (Figure 5C). This also indicates that DmDNA Ligase I fused to GFP is expressed and folded properly and that the inability to accumulate at RF in Drosophila cells cannot be attributed to improper expression or misfolding.

The absence of accumulation of both HsDNA Lig I and DmDNA Lig I at RF in Drosophila cells, and rather a diffused nuclear distribution, could be attributed to a high background of overexpressed protein that might occlude accumulation at RF. Such occluded accumulation could be then revealed by detergent extraction of soluble proteins [30]. However, the CMV-IE promoter used in all the expression vectors here is much weaker in Drosophila cells compared to the mammalian cells. The expression is about 10 times lower in Drosophila cells compared to mammalian cells as indicated by the observation that the intensity of GFP signal in Drosophila cells is 10 times less than that in mammalian cells. Thus, lower expression levels in Drosophila cells preclude masking of GFP-fusion protein accumulation at RF.

Interestingly, amongst the various PCNA-binding proteins tested only Dnmt1 and its deletion, NMT, showed some punctate distribution in the nucleus, which was though mostly excluded from BrdU stained regions. This punctate pattern was more evident with NMT whereas the GFP fusions of HsDNA Lig I and DmDNA Lig I showed more diffused nuclear pattern. In mammalian cells Dnmt1 shows a preference for heterochromatic regions via a domain in its N-terminus called TS during the G2 phase. Analysis of the punctate Dnmt1 pattern with Drosophila heterochromatin stained using anti-HP1 antibody did not reveal any preferential accumulation of Dnmt1 at Drosophila heterochromatin (data not shown). Thus, it is not completely clear what the punctate Dnmt1 pattern means. Further, this punctate pattern is more pronounced with NMT-GFP where the accumulation tends to be around the nucleolus in most cells (Figure 3C). One explanation for this accumulation could be that the N-terminal domain of Dnmt1 has multiple functional domains that have been show to interact with DNA and various proteins (like PCNA, HDAC, Rb, HP1, G9a) in mammalian cells. In mammalian cells these interactions are regulated, for example, we have shown that the interaction with PCNA is cell cycle dependent [7]. However, Dnmt1 being a completely foreign protein in Drosophila cells, these interactions or other non-specific interactions might not be regulated, which probably results in some aggregation.

The fact that except for PCNA, no localization at Drosophila RF was observed with the other replication factors analyzed here does not rule out very transient and short-lived associations of these factors with the RF. This is supported by recent studies from our group where clear differences in the mobility and interaction of PCNA binding proteins and PCNA with RF were measured. Whereas PCNA formed a stable accumulation at the RF, PCNA interacting proteins like DNA Ligase I and FenI only transiently associated and were not stable components of the replication machinery [31,32]. Thus, lack of accumulation of proteins at RF does not exclude interaction with components at RF. This could be true for studies on other proteins and their cognate cellular structures and lack of accumulation cannot be interpreted as absence of activity at the respective cellular structures.

## Conclusion

In conclusion, no accumulation of replication factors at RF could be detected in Drosophila cells, suggesting a fundamental difference between Drosophila and mammalian cells. While replication in Drosophila cells seems to rely on transient interaction with highly mobile factors, mammalian cells seem to have evolved more stable interactions with higher order structures possibly to cope with the challenges of replicating a genome 22 times as big as the fly genome.

## Methods

### Expression plasmids

The various plasmid constructs encoding translational fusions of Dnmt1 [7], HsPCNA [6], DmPCNA, HsDNA Ligase I [5] and DmDNA Ligase I with GFP were derived from the following vectors: pEMT [33], pEGFP-N2 (Clontech), pEGFP-C1 (Clontech), pEVRF0 [34]. Eucaryotic expression in all cases is driven by the cytomegalovirus immediate-early enhancer-promoter. Wherever PCR was used to generate the required insert, the sequence was verified by sequencing (GATC). The PBD inserts for MTPBD-GFP, HsLigPBD-GFP and DmLigPBD-GFP fusion proteins were generated from annealed sense and antisense oligonucleotides corresponding to PBD coding region (sequences available on request). The oligonucleotides were flanked with overhangs corresponding to products of *Eco *RI and *Xma *I digestions. The sense and anti-sense oligonucleotides were annealed in Sequenase buffer [0.2 M Tris-Cl (pH 7.5), 0.2 M MgCl _2_, 1 M NaCl] and subcloned into pEGFP-N2 digested with *Eco *RI and *Xma *I.

### Cell culture and transfections

*Drosophila *Schneider's line 2 (S2 cells) were grown in a humidified atmosphere at a temperature of 25°C and maintained in Shields and Sang M3 medium (Sigma) with 10% FCS at a density of 0.5 × 10^6 ^to 2 × 10^6 ^cells/ml. Transient transfections were done using the CaPO4-DNA coprecipitation method [35,36]. Cells were transfected at a density of 1 × 10^6 ^cells/ml. 48 hrs after transfection, the cells were washed gently with phosphate buffered saline (PBS), fixed with 3.7% formaldehyde in PBS and immunostained.

### BrdU labeling and immunofluorescence staining

For immunostaining and microscopic analysis, S2 cells were cultured on polylysine coated glass coverslips to enhance attachment of cells to the coverslip. For detection of RF, cells grown on coverslips were incubated in medium with 100 μM BrdU for 15 min (pulse labeling), washed twice with PBS and fixed with 3.7% formaldehyde in PBS. Cells were permeabilized with 0.25% Triton-X-100 for 10 min, washed twice with PBS and blocked in 0.2% fish skin gelatin (FSG) for 30 min. The cells were then incubated with mouse monoclonal anti-BrdU antibody (Beckton-Dickinson) or rat monoclonal anti-BrdU antibody (Harlan Seralab) along with other desired primary antibodies against replication proteins (DNA Ligase I, Dnmt1, PCNA) for 1 hr at 37°C. The primary antibodies were diluted in buffer containing 0.2% FSG, 20 U/ml DNase I (Boehringer Mannheim), 0.5 mM ßmercaptoethanol, 0.33 mM MgCl2, 33 mM Tris-Cl pH 8.1. The following primary antibodies were used in the various experiments: rabbit anti-PATH52 (against Dnmt1) [1: 2000; [[37]]], mouse monoclonal anti-PCNA (1:1000 with methanol fixed cells, Clone PC10, Dako), rabbit anti-PCNA (1:100, FL-261, Santa Cruz), rabbit anti-DNA Ligase I [1:250, [[5]]]. Fluorophore (FITC/Texas red/Cy5/Alexa Fluor 568/Alexa Fluor 647) conjugated secondary antibodies were used for detection. DNA was counterstained with Hoechst 33258 or TOPRO-3 and cells were mounted in mowiol with 2.5% DABCO as an anti-fade agent.

### Microscopy and image analysis

Immunostained cells were examined on a Zeiss LSM 510 microscope with 63× or 100× NA 1.4 Plan-Apochromat oil immersion objective with Nomarski optics. Ar-laser (488, 514 nm), HeNe-laser 1 (543 nm) and HeNe-laser 2 (633 nm) were used to excite the fluorophores. Images were acquired using the Zeiss LSM510 software and processed, assembled and annotated using Adobe Photoshop and Adobe Illustrator.

Colocalization analyses were performed on raw images after correcting for background using the JACoP plugin in ImageJ [38]. The average size of replication foci was determined by measuring the diameter (in the xy plane) of 7–10 RF per nuclei from 5 nuclei for each cell type using ImageJ tools.

### Search for DNA Ligase I homologue in Drosophila

DmDNA Ligase I cDNA was identified by searching the *Drosophila *genome database and the EST database (BDGP) [28] with DNA Ligase I sequences from different organisms (mouse, human, yeast) as query using the BLAST program [39]. The sequences obtained were aligned pairwise using BLAST2 [40] for generating the schematic of alignments shown in Figure 4A. Phylogenetic comparison of the human DNA Ligases and the putative *Drosophila *Ligases were done using the Jotun-Hein method [41] in the Lasergene program. The DmDNA Ligase I cDNA clone (LD41868) was obtained from ResGen. Multiple sequence alignments were generated using the Lasergene software.

## List of abbreviations

PCNA: proliferating cell nuclear antigen; PBD: PCNA binding domain; HsDNA Lig I: Homo sapiens DNA Ligase I; DmDNA Lig I: Drosophila melanogaster DNA Ligase I; HsPCNA: Homo sapiens PCNA; DmPCNA: Drosophila melanogaster PCNA; ScPCNA: Saccharomyces cerevisiae PCNA; RFTS: replication foci targeting sequence

**Abbreviations used to denote various fusion proteins: **NMT-GFP: N-terminal domain of Dnmt1 fused to GFP; MTPBD: PBD domain from Dnmt1 fused to GFP; HsLigPBD-GFP: PBD domain from HsDNA Lig I fused to GFP; DmLigPBD-GFP: PBD domain from DmDNA Lig I fused to GFP; GdL: DmDNA Lig I fused to GFP.

## Authors' contributions

HPE participated in the design of the study, carried out the experiments and data analysis and wrote the manuscript. HL conceived the study and helped to draft the manuscript. MCC conceived the study, participated in its design and coordination and wrote the manuscript. All authors have read and approved the final manuscript.
